# Inhibition of Lysyl Oxidase and Lysyl Oxidase-Like Enzymes Has Tumour-Promoting and Tumour-Suppressing Roles in Experimental Prostate Cancer

**DOI:** 10.1038/srep19608

**Published:** 2016-01-25

**Authors:** Maria Nilsson, Hanibal Adamo, Anders Bergh, Sofia Halin Bergström

**Affiliations:** 1 Department of Medical Biosciences, Pathology, Umeå University, Umeå, Sweden

## Abstract

Lysyl oxidase (LOX) and LOX-like (LOXL) enzymes are key players in extracellular matrix deposition and maturation. LOX promote tumour progression and metastasis, but it may also have tumour-inhibitory effects. Here we show that orthotopic implantation of rat prostate AT-1 tumour cells increased LOX and LOXLs mRNA expressions in the tumour and in the surrounding non-malignant prostate tissue. Inhibition of LOX enzymes, using Beta-aminopropionitrile (BAPN), initiated before implantation of AT-1 cells, reduced tumour growth. Conversely, treatment that was started after the tumours were established resulted in unaffected or increased tumour growth. Moreover, treatment with BAPN did not suppress the formation of spontaneous lymph node metastases, or lung tumour burden, when tumour cells were injected intravenously. A temporal decrease in collagen fibre content, which is a target for LOX, was observed in tumours and in the tumour-adjacent prostate tissue. This may explain why early BAPN treatment is more effective in inhibiting tumour growth compared to treatment initiated later. Our data suggest that the enzymatic function of the LOX family is context-dependent, with both tumour-suppressing and tumour-promoting properties in prostate cancer. Further investigations are needed to understand the circumstances under which LOX inhibition may be used as a therapeutic target for cancer patients.

The Lysyl oxidase (LOX) family has five members, LOX and Lysyl oxidase-like (LOXL) 1–4, which are all secreted copper-dependent amine oxidases with the primary function to covalently cross-link collagen and elastin in the extracellular matrix (ECM), though intracellular functions of LOX have also been reported^1^,^2^,^3^. The role of the LOX family in cancer is unclear, and the data available (dealing particularly with LOX and LOXL2^4^) suggest that these enzymes have both tumour-suppressing and tumour-promoting effects. Increased activity of extracellular LOX remodels the ECM and creates a stiffer microenvironment that promotes tumour progression and metastasis^5^,^6^. LOX promotes tumour cell invasion^6^,^7^,^8^, and is required for pre-metastatic niche formation^9^. LOX secreted by breast cancer cells, has been shown to induce pre-metastatic bone lesions that precedes and facilitates the formation of bone metastases^10^. Inhibition of LOX has been found to suppress the establishment of lung and liver metastases in an orthotopic breast cancer model^6^. Recently, LOX was shown as a predictive marker in pancreatic cancer, and inhibition of LOX in mouse models of pancreatic tumours reduced metastases and affected primary tumours in combination with gemcitabine^8^. Tumour-secreted LOXL2 activates fibroblasts and induces collagen remodelling in the ECM^11^. Furthermore, LOXL2 has been shown to promote tumour growth and metastasis in a breast cancer model^12^,^13^, and is critical for niche formation in hepatocellular carcinoma^14^. LOXL4 increases proliferation and metastasis in gastric cancer^15^, and silencing *Loxl4* expression in bladder cancer using miRNA-193a-3p retards tumour growth^16^. These results have implicated LOX, LOXL2 and LOXL4 as possible therapeutic targets, particularly for metastatic disease. However, other studies have suggested that the LOX enzymes suppress tumours. LOX inhibits HRAS-induced tumour formation and reverses HRAS transformation of fibroblasts^17^,^18^. LOX is secreted as a proenzyme and it is subsequently cleaved into catalytically mature LOX and a LOX propeptide (LOX-PP)^2^,^19^,^20^. The LOX-PP has tumour-suppressing properties^21^,^22^,^23^. Reduced LOX expression has been observed in a number of cancers, and has implicated *LOX* as a tumour suppressor gene^24^,^25^,^26^. It has also been suggested that LOXL1 and LOXL4 may have tumour-suppressing roles in bladder cancer^27^.

The role of the LOX family in prostate tumour progression is largely unknown. High expression of *LOX* mRNA is associated with high-grade prostate tumours and tumour recurrence^28^, and has been shown to correlate with a high Gleason score^29^. Conversely, *LOX* mRNA expression has been found to be lower in metastatic prostate tumours than in primary prostate tumours^30^, and low levels of LOX expression have also been reported in high-grade tumours^31^,^32^, suggesting loss of LOX expression during prostate tumour progression. Reduced LOXL2 expression has been shown in prostate tumours due to deletion of the chromosome region containing the *LOXL2* gene^33^. Using immunohistochemistry, *in situ* hybridization and transcriptomics we showed that LOX was synthesized both in tumour epithelial cells and in epithelial cells in the non-malignant normal parts of the tumour-bearing organ^34^. High LOX levels in the tumour epithelium correlated to high Gleason score and presence of metastases at diagnosis, and high LOX in the normal epithelium was associated with a poor outcome and gave prognostic information in addition to tumour Gleason score^34^. Implantation of rat prostate cancer cells into the rat prostate resulted in increased expression of LOX in the tumour and in the rest of the tumour-bearing prostate lobe^35^ suggesting that tumours may stimulate LOX synthesis in adjacent tissues.

The aim of this study was to investigate the expression of LOX and LOXL enzymes in orthotopic rat prostate tumours and in the tumour-adjacent normal prostate tissue, and how Beta-aminopropionitrile (BAPN) treatment (an irreversible inhibitor of LOX and other LOX-family members’ catalytic activity^36^,^37^,^38^,^39^,^40^,^41^) would affect rat prostate tumour growth and metastasis. BAPN treatment initiated prior to orthotopic tumour cell implantation resulted in significantly reduced tumour growth, whereas BAPN treatment started after tumour establishment significantly increased tumour growth. BAPN treatment did not affect spontaneous formation of lymph node metastases. Similarly, no effect on lung tumour burden was found when the tumour cells were injected intravenously. Our results suggest that the enzymatic function of LOX is highly context-dependent and that it can have both tumour-suppressing and tumour-promoting properties in prostate cancer.

## Results

### Expression of the LOX family in orthotopic rat prostate tumours and in the surrounding normal prostate tissue

In a recent microarray study we found that that the expressions of *Lox*, *Loxl1*, and *Loxl2* increased in orthotopic rat prostate AT-1 tumours and in the tumour-adjacent normal prostate tissue compared to tumour-free control rat prostate tissue, suggesting that these enzymes may have a role in tumour progression or tumour defence in prostate cancer^35^.

**Figure 1:**
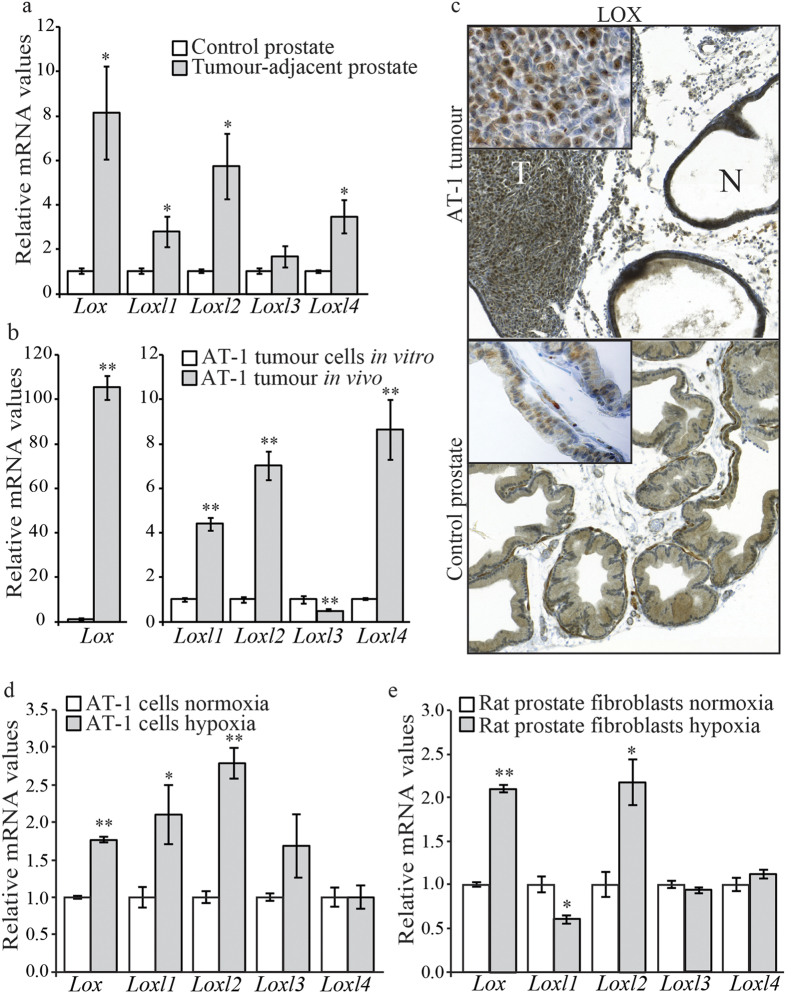
Expression of *Lox* and *Loxl1—Loxl4* mRNAs in orthotopic rat prostate tumours and in the surrounding normal rat prostate tissue. (**a**) Relative mRNA expression of *Lox* and *Loxl1—Loxl4* in non-malignant tumour-adjacent rat prostate tissue (*n* = *8*) expressed in relation to the levels of each factor in tumour-free control rat prostate tissue (*n* = *7*) (**b**) Relative mRNA expression of *Lox* and *Loxl1-Loxl4* in orthotopic AT-1 tumours (*n* = *8*) on day 10 after tumour implantation, expressed in relation to the levels of each factor in the AT-1 tumour cells *in vitro*. (**c**) Representative sections from a 10-day orthotopic AT-1 rat prostate tumour (T), tumour-adjacent normal rat prostate tissue (N), and tumour-free control rat prostate tissue stained for LOX (inserts show LOX staining in higher magnifications). (**d**,**e**) Relative mRNA expression of *Lox* and *Loxl1-Loxl4* in rat prostate AT-1 tumour cells (**d**) and in primary rat prostate fibroblasts grown *in vitro* (**e**) under normoxic or hypoxic conditions for 24 h. Values are mean ± SEM; *p < 0.05, **p < 0.01.

To verify these results, AT-1 tumour cells were injected into the prostate of fully immunocompetent Copenhagen rats and grown for 10 days and the mRNA expressions of *Lox* and *Loxl1-Loxl4* were examined using qRT-PCR. As found previously, the expression of *Lox*, *Loxl1, Loxl2*, and also *Loxl4*, was significantly higher in the tumour-adjacent non-malignant prostate tissue than in tumour-free vehicle injected control prostate tissue (Fig. 1a), showing that the presence of a tumour can induce the expression of *Lox* and *Loxl* in the tumour-bearing organ. In addition, we compared mRNA expression of *Lox* and *Loxl1-Loxl4* in AT-1 tumours *in vivo* with that in AT-1 tumour cells *in vitro*. All genes encoding the LOX family, except *Loxl3*, had significantly higher mRNA expression in the tumour tissue *in vivo* than in the tumour cells *in vitro* (Fig. 1b). The highest relative increase in mRNA expression in the tumours was seen for *Lox*, whereas *Loxl3* mRNA expression was significantly reduced in implanted AT-1 tumours. Taken together, these results suggest that some factor or factors in the prostate microenvironment up-regulate the expression of LOX enzymes both in prostate tumour tissue and in the surrounding normal prostate tissue.

In addition, we examined LOX protein expression using immunohistochemistry. In AT-1 tumours, LOX expression varied within the tumours—with both nuclear and cytoplasmic staining (Fig. 1c). This type of tumour grows in a poorly developed stroma, and LOX expression in the tumour stroma was therefore difficult to evaluate. LOX expression was detected in glandular epithelial cells and in stromal smooth muscle cells in both tumour-adjacent non-malignant prostate tissue and tumour-free normal rat prostate tissue (Fig. 1c). The intensity of LOX immunoreactivity was, however, not significantly different in the tumour-adjacent prostate tissue and in tumour-free prostate tissue.

### Expression of LOX family members are increased by hypoxia

We have shown in previous studies that orthotopic AT-1 tumours are hypoxic and that the tumour-adjacent rat prostate tissue is more hypoxic than tumour-free prostate tissue^35^,^42^. As the expression of LOX, LOXL1, LOXL2, and LOXL4 was increased by hypoxia in other tissues^6^,^43^,^44^,^45^,^46^, we investigated whether hypoxia would induce the expression of LOX enzymes in prostate cancer. AT-1 cells incubated under hypoxia for 24 h *in vitro* had significantly higher *Lox*, *Loxl1*, and *Loxl2* mRNA levels than normoxic controls (Fig. 1d). To determine whether hypoxia also could induce the expression of LOX enzymes in non-malignant rat prostate, we prepared primary cultures of rat prostate fibroblasts *in vitro*. We could not, however, study LOX expression in rat prostate epithelial cells since we were unable to isolate these cells in culture. Hypoxia increased the mRNA expression of *Lox* and *Loxl2* in primary rat prostate fibroblasts (Fig. 1e). This suggests that upregulation of these LOX-family enzymes in both the tumour tissue and non-malignant rat prostate tissue could be due to hypoxia.

### BAPN treatment of rats with orthotopic prostate tumours, and in a lung colonization model

**Figure 2:**
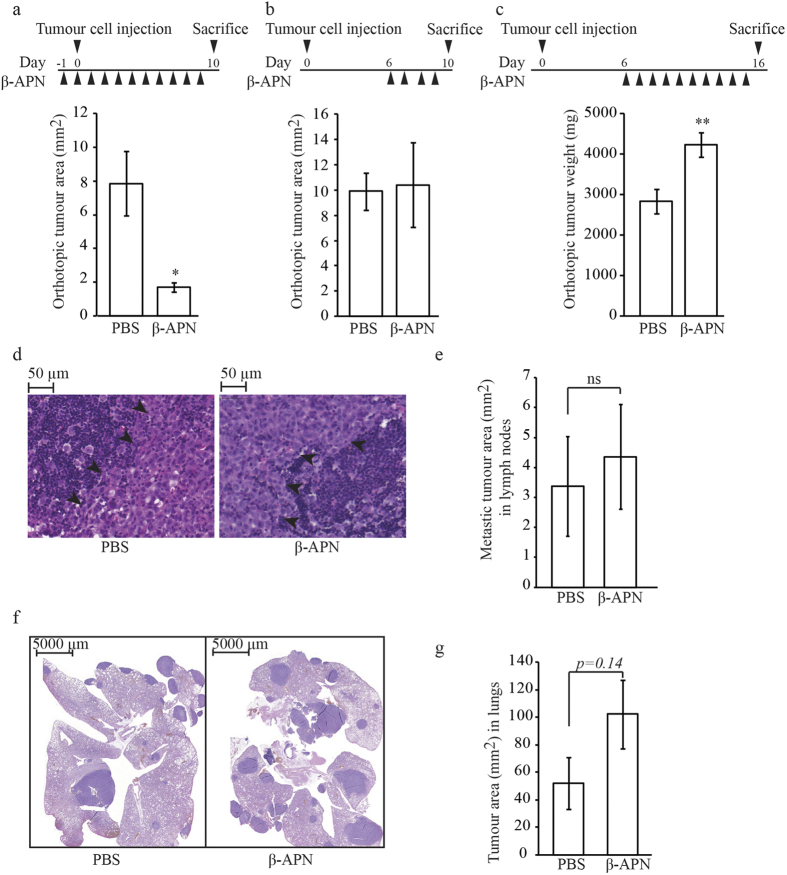
BAPN treatment of rat prostate tumours. Rats were injected orthotopically with AT-1 prostate tumour cells (2 × 10^3^ cells) and treated with an inhibitor of LOX-family enzymes (BAPN, 100 mg/kg) or vehicle (PBS) and tumour weight was analyzed in three different experiments: (**a**) treatment was started one day before tumour cell implantation and continued on a daily basis until sacrifice at day 10; (**b**) treatment was started on day 6 when tumours were established, and continued on a daily basis until sacrifice at day 10; and (**c**) treatment started on day 6 and continued on a daily basis until sacrifice at day 16. Values are mean tumour area (mm^2^) or mean tumour weight (mg) at sacrifice ± SEM; *n* = *6–8 per group*; *p < 0.05, **p < 0.01. (**d**) Representative haematoxylin- and eosin-stained sections showing tumour growth in the lymph nodes of both PBS- and BAPN-treated animals (arrowheads indicate the border between lymph node tissue and tumour tissue). (**e**) Tumour area of spontaneous lymph node metastases in animals with orthotopic tumours treated with BAPN or PBS from day 6 to day 16. Values are mean ± SEM; *n* = *5 per group;* ns: not significant (**f**) Representative haematoxylin- and eosin-stained sections showing tumour growth in the lungs of both PBS- and BAPN-treated animals. (**g**) Lung tumour burden in animals injected intravenously with AT-1 cells and treated with a LOX inhibitor (BAPN, 100 mg/kg) or vehicle (PBS) from day 6 to day 16. Values are mean ± SEM; *n* = *7 per group*.

Targeting of the LOX family has been suggested as a new cancer therapy^4^. The effect of such a treatment for prostate cancer has not been explored. To examine this further, we treated rats with AT-1 tumours with BAPN, an irreversible inhibitor of LOX and other LOX-family members^36^,^37^,^38^,^39^,^40^,^41^. When BAPN treatment was started one day before tumour cell implantation and continued until sacrifice at day 10, AT-1 tumour size was significantly reduced (by 80%) compared to PBS-treated controls (Fig. 2a). If, however, BAPN treatment was started on day 6—when tumours were already established—and continued until day 10, tumour growth was unaffected (Fig. 2b). Surprisingly, when BAPN treatment was started on day 6 and continued until day 16, AT-1 tumour weight increased significantly (by 50%) compared to the PBS-treated controls (Fig. 2c). Our results suggest that the enzymatic activities of LOX proteins are important for tumour progression during the early stages of tumour establishment, as BAPN treatment at this point inhibited tumour growth. Conversely, at later time points—when the tumours have already been established—the enzymatic activity instead appears to have tumour-suppressing roles, as BAPN administration resulted in increased tumour growth. This illustrates the complex and somewhat paradoxical roles of LOX enzymes during tumour progression.

To determine whether BAPN treatment had any effect on metastatic ability, the animals with orthotopic tumours were examined for the presence of lymph node and lung metastases. No metastases were detected in the lymph nodes or lungs 10 days after tumour cell implantation. Although AT-1 is not a highly metastatic tumour, we detected spontaneous lymph node metastases in both BAPN treated animals (5/7) and control animals (5/6) on day 16 after intra-prostatic implantation of tumour cells (Fig. 2d). In addition, there was no significant difference in tumour area in lymph nodes of treated animals compared to control animals (Fig. 2e). Tumour tissue occupied approximately 30% of the total lymph node area in both groups (PBS; 28 +/− 16 vs. BAPN; 30 +/− 12%, mean +/− SEM, p = 0.602) and there was no significant difference in the total lymph node area analyzed for each group (PBS; 12.9 +/− 1.7 vs. BAPN; 14.3 +/− 0.6 mm^2^, p = 0.754). This shows that BAPN treatment did not affect metastatic capacity in the model tested. Only one animal in the control group and two animals in the BAPN-treated group had microscopic metastatic lesions in their lungs at this time point.

To simulate metastatic colonization in the lungs, AT-1 tumour cells were injected in the tail vein and BAPN treatment was started on day 6 and continued on a daily basis until day 16. There was no significant difference between lung tumour burden in BAPN-treated animals and that in the controls (Fig. 2f,g), and only small differences were found in the mean number of tumour foci (32 for BAPN treatment vs. 27 for PBS). Although not significant, lung tumour burden was actually greater in the BAPN-treated animals than in the controls. This suggests that care should be taken before inhibiting LOX in the metastatic setting, and that more studies are needed to examine the function of LOX in the metastatic microenvironment.

### Viability of AT-1 tumour cells treated with BAPN or recombinant LOX protein *in vitro*

**Figure 3:**
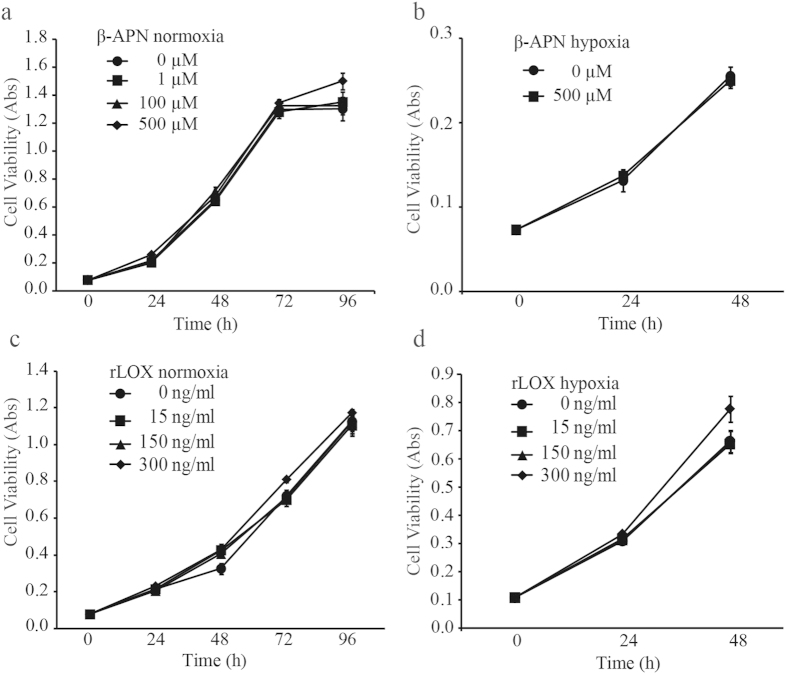
Cell viability of AT-1 tumour cells treated with BAPN or recombinant LOX *in vitro*. Cell viability was measured at different time-points using an MTT assay of cultured AT-1 cells incubated with different concentrations of (**a**) BAPN in normoxia, (**b**) BAPN in hypoxia, (**c**) recombinant LOX protein in normoxia, and (**d**) recombinant LOX in hypoxia. No significant changes were found.

To determine whether BAPN treatment directly affected tumour cell growth, we examined how BAPN affected AT-1 cell viability *in vitro* under both hypoxic and normoxic conditions. BAPN treatment did not affect tumour cell viability under normoxic or hypoxic conditions compared to untreated controls (Fig. 3a,b). In addition, we treated the AT-1 cells *in vitro* with increasing concentrations of recombinant LOX protein under both normoxia and hypoxia to determine whether LOX could affect tumour cell viability directly. LOX did not affect AT-1 tumour cell viability at any of the concentrations or under any of the conditions used (Fig. 3c,d). As neither inhibition of LOX enzymatic activity with BAPN nor stimulation with recombinant LOX protein affected AT-1 viability *in vitro*, LOX does probably not directly affect tumour cell viability. The effects of BAPN treatment on tumour growth seen in our animal model are therefore probably due to LOX activities in the tumour stroma and/or the surrounding tumour-adjacent non-malignant prostate stroma and epithelium.

### Collagen fibre content and expression of factors related to ECM remodelling in the AT-1 tumour and in the tumour-adjacent prostate tissue

**Figure 4:**
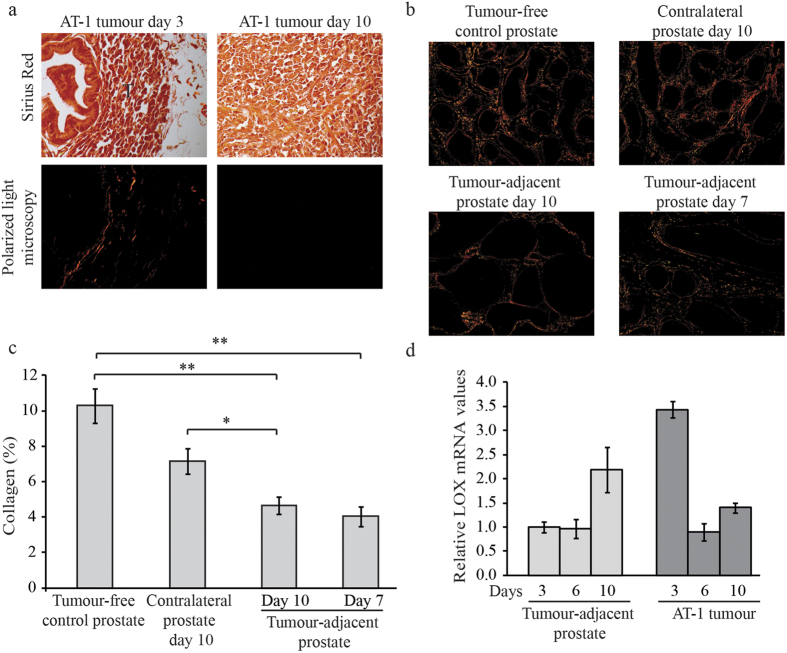
Collagen fibre density in AT-1 tumours and in tumour-adjacent non-malignant prostate tissue. (**a**) Representative sections of AT-1 tumours at day 3 and day 10 stained with Sirius red in both regular light microscopy and in polarized light microscopy (Original magnifications 400x, T; tumour). (**b**) Representative sections of tumour-adjacent prostate tissue at day 7 and 10, contralateral prostate tissue at day 10 and tumour-free control prostate tissue stained with Sirius red and shown in polarized light. (**c**) Quantification of Sirius red staining in tumour-adjacent prostate tissue and controls. Values are the mean collagen percentage ± SEM; *n* = *5–7 per group*; *p < 0.05, **p < 0.01. (**d**) Relative mRNA expression of *Lox* and in AT-1 tumours and in non-malignant tumour-adjacent rat prostate tissue at 3, 6 and 10 days after tumour cell implantation (*n* = *2*–*8 per group*) expressed in relation to the levels in tumour-adjacent prostate tissue at day 3.

As LOX cross-links collagen and elastins, we stained our prostate samples with Sirius red and examined the sections under polarized light. Small AT-1 tumours at day 3 contained collagen fibres while almost no collagen was found inside AT-1 tumours at day 7 or 10 (Fig. 4a and data not shown. This demonstrates that the extracellular target for LOX was only present in the tumour stroma during early tumour stages in this model and almost absent within the tumour stroma at later time points. This could explain why LOX inhibition was more effective when initiated earlier. Furthermore, the density of Sirius red stained fibres in the tumour-adjacent non-malignant prostate tissue at day 10, measured 0–2 mm out from the tumour border, was significantly lower than in the contralateral prostate lobe and compared to tumour-free controls (Fig. 4b,c), suggesting that the presence of a tumour results in loss of collagen fibres not only in the tumour stroma but also outside in the stroma of the tumour-bearing organ. This loss of collagen in the surrounding non-malignant tissue was also observed at day 7 (Fig. 4c), suggesting that collagens were reduced in the tumour-adjacent tissue when LOX inhibition was initiated for the established tumours. Taken together this shows that the target for LOX was absent in the stroma of established tumours and reduced in the surrounding non-malignant tissue, which may explain why BAPN treatment did not inhibit tumour growth at later time points. Moreover, LOX mRNA expression was higher in AT-1 tumours at day 3 compared to at day 6 and 10 while LOX mRNA expression in the tumour-adjacent non-malignant prostate tissue was increased at day 10 compared to the earlier time points (Fig. 4d). This suggests that LOX was more active in small tumours that contained more collagen, and inhibiting LOX using BAPN at this time point may therefore suppress tumour growth. Conversely, at later time points inhibiting LOX may affect other aspects of LOX than collagen cross-linking, that possibly could enhance tumour growth.

Hypoxia has been suggested to stimulate collagen degradation in tumours^47^. Increased LOX expression in the tumour-adjacent non-malignant tissue at day 10 could thus be part of an ECM remodelling response in the tumour-bearing organ. We therefore further examined our previously published data on global gene-expression^35^ in AT-1 tumours and the surrounding non-malignant tissue at day 10 for more changes in factors related to ECM remodelling. Particular interest was given targets for LOX and LOX-family members such as collagens and elastin, and factors affecting their levels such as matrix metallo-proteinases (*Mmp*), their inhibitors (*Timp*) and other proteases. Expression-levels changed more that 2-fold compared to tumour-free (vehicle injected) normal prostate tissue controls and with a p-value <0.05 were considered. In tumours, *Collagen 1a1* (5.0 fold), *Collagen 3a1* (5.0 fold), *Collagen 4a1* (2.4 fold), *Collagen 4a3* (2.1 fold), *Collagen 5a1* (4.6 fold), *Collagen 5a2* (11 fold), *Collagen 18a1* (7 fold), *Mmp3* (21 fold) *Mmp14* (4.5 fold), *Timp1* (2.5 fold), *Cathepsin L* (2.4 fold), *Cathepsin S* (2.3 fold), *uPa* (2-fold), *Prolyl 4-hydroxylase alpha 1* (*P4Ha1*)(3.6 fold) and *P4hb* (−1.7 fold) were altered compared to controls^35^. In the tumour-adjacent non-malignant prostate tissue, *Collagen 1a1* (4 fold), *Collagen 1a2* (2.4 fold), *Collagen 3a1* (5 fold), *Collagen 4a1* (3.8 fold), *Collagen 5a1* (3 fold), *Collagen 5a2* (3.8 fold), *Collagen 15a1* (2.5 fold), *Collagen 28a1* (2.2 fold), *Mmp3* (2.6 fold), *Mmp14* (2.7 fold), *Timp1* (3.3 fold), *Elastin* (3.4 fold), *Cathepsin L* (1.8 fold), *Cathepsin S* (2.4 fold), *uPa* (2 fold), *P4ha1* (1.8 fold) showed changed expression compared to control prostate tissue^35^. This suggests that both the tumour and the tumour-adjacent non-malignant prostate tissue have active ECM remodelling, with a net balance between collagen synthesis and degradation that favours degradation.

## Discussion

LOX has been shown to be an important regulator of the ECM, for pre-metastatic niche formation, and for metastatic establishment and growth of metastases^5^,^6^,^8^,^9^,^10^. However, LOX and LOX by-products have also been shown to have tumour-suppressor functions^17^,^18^,^22^,^23^,^26^. We therefore require a better understanding of these phenomena in order to identify tumours that are suitable for anti-LOX treatment and in what context, and also the circumstances under which LOX functions as a tumour suppressor. In the present study we used BAPN, an irreversible inhibitor of the catalytic activity of LOX and LOXL enzymes^36^,^37^,^38^,^39^,^40^,^41^, to study the function of the LOX family during prostate tumour progression. We have shown for the first time that treatment with BAPN resulted in opposite effects on prostate tumour growth, depending on when the treatment was initiated. When treatment was started one day before the orthotopic tumour cell implantation, this significantly reduced tumour growth—while starting the same treatment after tumour establishment increased tumour growth. Furthermore, BAPN treatment did not inhibit spontaneous metastatic growth in the lymph nodes and did not suppress lung tumour burden when the tumour cells were injected intravenously. The reasons behind these seemingly contradictory results are unknown, but they show that the role of LOX catalytic activity during tumour progression is complex. In a breast cancer model, inhibition of LOX reduced the formation of liver and lung metastases but did not affect orthotopic tumour growth^6^. When using the same type of breast tumour cells, BAPN treatment was found to reduce metastatic tumour burden in mice when the treatment was started one day before intra-cardiac tumour cell injection, but it had no effect on metastatic tumour growth when treatment was started on day 7^48^. Miller *et al*. showed that metastatic formation was inhibited when treating mice with pancreatic cancer with a LOX-blocking antibody, while no survival effect was shown with the antibody alone, indicating that the primary tumour was unaffected by the treatment^8^. In the same study, blocking LOX in combination with gemcitabine reduced metastases and increased survival of the mice when treatment was started on respectable tumours but not at later stages^8^. Together with our results, this indicates that LOX inhibition could be effective in preventing tumour initiation and metastatic colonization. Using data from our previously published study we note that the expression of collagens (targets for LOX) but also *Mmp*s and other proteases, were increased in tumour and in tumour-adjacent non-malignant prostate tissue in rats^35^. Sirius red staining showed almost no collagen inside larger tumours, possibly due to simultaneous increase in collagen degradation, but considerably more collagen was observed in the invasive zone and in the rest of the prostate lobe. This shows that the main target for extracellular LOX activity in established tumours is probably not within but outside tumours. A stimulatory effect of LOX on tumour establishment and growth could be particularly important early after tumour cell injection when few cells have to interact successfully with a potentially hostile collagen rich environment but perhaps less important for established tumours facing less collagen. Interestingly growth of the poorly differentiated and locally aggressive and hypoxic^35^ AT-1 tumour resulted in decreased Sirius red staining also in the tumour-adjacent non-malignant stroma possibly because both the tumour and the surrounding prostate tissue secreted factors degrading collagen fibres. We therefore suggest that the tumour promoting role of LOX in the tumour microenvironment decreases with time in tumour-types with a high capacity to stimulate degradation of the ECM. The low collagen content inside the tumours may also explain why there was no effect of the LOX treatment on metastasis in this model. One factor stimulating collagen degradation in tumours is hypoxia, and it has been suggested that once tumours become hypoxic the tumour cells become less dependent on a collagen matrix^47^.

In patients we have noted that high LOX in tumour epithelial cells was associated with high Gleason score but not with survival, whereas high LOX in non-malignant prostate epithelial cells was associated with a poor outcome^34^. The results in this study suggest that LOX levels should perhaps be related to collagen content in tumours and the tumour-bearing organ in order to elucidate its role in individual patients. In prostate cancer patients, tumour collagen content is apparently inversely related to tumour grade, but in the surrounding tissue it could be increased in cases with high grade tumours^49^, while collagen 1 content in tumour stroma is not associated with outcome^50^.

In this study treatment of established tumours with BAPN increased tumour growth. The reason to this is unknown but one possible explanation could be that increased LOX activity in the tumour-bearing organ, in contrast to the early tumour-promoting role in the tumour microenvironment, could be part of an insufficient defence trying to seal off the tumour. Inhibition of this defence in situations where the tumour-promoting role of LOX within the tumour is of limited importance may therefore actually speed tumour growth. Alternatively, in tumours with low collagen content, other tumour suppressing functions of LOX may be dominating. Further studies are needed to study these possibilities.

The LOXL proteins have not been thoroughly studied in tumourigenesis. If expressed, they are likely to have functions similar to those of LOX, both as tumour suppressors and tumour promoters^13^,^51^,^52^,^53^. This adds to the complexity of targeting the LOX family in cancer. We found that the mRNA expression of all members of the LOX family—except *Loxl3*—were up-regulated both in the tumour tissue and in the normal prostate tissue adjacent to the tumour. Thus, remodelling of the ECM by all of the LOX enzymes, by cross-linking collagen, might prepare the tumour-adjacent normal prostate tissue for tumour expansion and invasion or as suggested also be part of a defence mechanism induced to repair and strengthen the normal tissue. Further studies are needed to determine whether the different LOX enzymes have different functions in tumours or if they are able to compensate for each other. Whether or not there is a difference in the functions of these enzymes in tumours relative to their functions in the surrounding tissues is also unknown, and should be investigated further. As all LOX-family enzymes, except LOXL3, are upregulated under conditions of hypoxia^6^,^43^,^44^,^45^,^46^, hypoxia is likely to be one mechanism involved in the increased expression in our tumour model. Hypoxic incubation of AT-1 tumour cells *in vitro* increased the mRNA expression of *Lox*, *Loxl1*, and *Loxl2*, suggesting that hypoxia also increased their expression *in vivo*. Hypoxia is also likely to upregulate the expression of LOX-family enzymes in the normal prostate tissue, especially *Lox* and *Loxl2*, as hypoxia increased their mRNA expressions in rat prostate fibroblasts *in vitro*.

Intracellular localization of LOX has been observed in several cell lineages, suggesting that LOX may also have enzymatic activity inside cells^1^,^2^,^3^. Recently, intracellular expression of *Loxl2* in a breast cancer model stimulated tumour invasiveness^54^. Whether tumour-suppressive vs. tumour-enhancing functions, are due to temporal changes in the relative importance of intracellular vs. extracellular LOX activity, different roles of LOX in the intra-tumoural vs. extra-tumoural environments, as well as temporal changes in available collagens and elastin to cross-link is unknown, and warrants further investigation. The role of LOX as a tumour suppressor has been proposed as an intracellular function, mainly mediated via the LOX-PP^4^,^21^. Treatment with BAPN or stimulation with recombinant LOX peptide did not affect tumour cell viability *in vitro*, suggesting that LOX does not affect the AT-1 tumour cells directly. The effects of BAPN treatment *in vivo* are therefore probably due to inhibition of LOX activities in the tumour stroma and in the surrounding prostate tissue.

In summary, functional studies inhibiting LOX enzymes in an orthotopic prostate cancer model resulted in opposite effects, with reduced tumour growth if the treatment was initiated before to tumour cell implantation, and increased tumour growth if the treatment was initiated when tumours were already established. Anti-LOX treatment did not affect the growth of metastases in lymph nodes or lungs. The paradoxical roles of the LOX family therefore need to be studied further, to enhance the tumour-suppressive effects while inhibiting the tumour-promoting ones.

## Materials and Methods

### Animal studies and treatments

Dunning R-3327 AT-1 rat prostate tumour cells (ATCC, tested negative for murine viruses and mycoplasma with Impact Profile VIII, IDEXX Radil) were grown in culture as previously described^55^. AT-1 cells (2 × 10^3^ in 10 μl RPMI 1640) or vehicle (10 μl RPMI 1640) were carefully injected into one lobe of the ventral prostate of adult male Copenhagen rats (Charles River, Sulzfeld, Germany; bred in our laboratory) as previously described^42^. The animals were sacrificed 3, 6, 7 and 10 days later, when the tumours were still surrounded by non-malignant prostate tissue, and the prostate lobes were quickly removed and snap-frozen and/or formalin-fixed for further analysis.

For LOX inhibition studies, AT-1 cells (2 × 10^3^) were injected into rat prostate and the rats were treated with intraperitoneal injections of the LOX inhibitor BAPN (100 mg/kg, 0.39 M; Sigma-Aldrich) or PBS (matching controls). Three different experiments were performed for orthotopic tumours: (1) treatment started one day before to tumour cell injection and continued on a daily basis until sacrifice on day 10 (*BAPN, n* = *8; PBS, n* = *8*); (2) treatment started on day 6 continued on a daily basis until sacrificed on day 10 (*BAPN, n* = *8; PBS, n* = *8*); and (3) treatment started on day 6 and continued on a daily basis until sacrifice on day 16 (*BAPN, n* = *7; PBS, n* = *6*). The prostate lobes containing the tumours were removed, weighed, formalin-fixed and paraffin-embedded. The lymph nodes and lungs were removed and formalin-fixed to study spontaneous formation of metastases. Furthermore, AT-1 cells (2.5 × 10^6^ cells, 250 μl) were injected in the tail vein to simulate metastatic colonization in the lungs, and treatment started on day 6 and continued on a daily basis until sacrifice on day 16 (*BAPN, n* = *7; PBS, n* = *7*). For small orthotopic tumours growing within the ventral prostate tissue (treatment from day -1 to 10 and day 6–10), tumour area was assessed in haematoxylin- and eosin-stained prostate tissue sections using the Pannoramic Viewer software version 1.15 (3DHistech, www.3dhistech.com). The section with the largest tumour area in each animal was chosen to represent tumour size. For large orthotopic tumours that had outsized the normal prostate tissue and predominantly contained tumour tissue (treatment from day 6–16), tumor weight represent tumor size. For lymph node metastases, total tumour area in all lymph node tissue in each animal was analyzed on representative sections and represented the lymph node tumour size. For tumours in the lungs, tumour size is given as the total area of all tumours in a representative section of each lung. Animal experiments were carried out in accordance with protocols approved by the Umeå Ethical Committee for animal studies (permit number A110-12).

### RNA preparation and quantitative RT-PCR analysis

Frozen prostate tissues, containing both AT-1 tumour and surrounding non-malignant tissue, were sectioned and stained with haematoxylin and eosin to determine tumour location. Using the sections for guidance, frozen tumour tissue and surrounding non-malignant tissue were separated and dissected with a surgical blade. To avoid contamination with tumour tissue, the non-malignant tissue was taken with a spacing of 1 – 2 mm from the tumour. Total RNA was then extracted from AT-1 tumour tissue (day 3; *n* = 2, day 6; *n* = 4, day 10; *n* = *8*), corresponding adjacent non-malignant prostate tissue (day 3; *n* = 5, day 6; *n* = 4, day 10; *n* = *8*), tumour-free vehicle-injected control prostate tissue (*n* = *7*), and AT-1 cells *in vitro* (*n* = *2 different cell culture flasks*), using the TRIzol method according to the manufacturer’s directions (Invitrogen). The RNA was DNase-treated (DNase 1; Ambion) to remove contaminating DNA, and 0.8 μg was used for synthesis of cDNA using Superscript II according to the manufacturer’s instructions (Invitrogen). Real-time qRT-PCR was performed using the Applied Biosystems 7900HT Real-Time PCR System and Taqman Gene Expression Assay (Applied Biosystems). Quantification of mRNA levels was done in a 20-μl reaction volume with 20 ng cDNA per reaction, with commercially available primer and probes (LOX, Rn005566984_m1; LOXL1, Rn01410838_m1; LOXL2, Rn01466080_m1; LOXL3, Rn01765242_m1; LOXL4, Rn01410872_m1) (Applied Biosystems). Negative controls were run in parallel, and the relative values for each gene were normalized using beta-actin (Rn00667869_m1) as reference gene. We used Taqman Analysis Software SDS2.4 (Applied Biosystems).

### LOX immunohistochemistry and Sirius Red staining

Rat prostate tissues were stained for LOX using the automatic staining system Ventana Benchmark Ultra (Ventana Medical Systems Inc.). Briefly, 4-μm-thick paraffin sections were pretreated with CC1 for antigen retrieval and stained with primary polyclonal antibodies to LOX (NB100-2527, diluted 1:100; Novus Biologicals). Samples were visualized using the ultraView Universal DAB Detection Kit. Sections were analysed using Pannoramic Viewer software. To visualize collagen fibres, paraffin sections from AT-1 tumours at day 7 (*n* = 5) and 10 (*n* = 7), and tumour-free controls (*n* = 5) were stained with Sirius red and examined by polarization microscopy^56^. The density of Sirius red stained fibres was assessed in 3 photos/animal/group and quantified using Photoshop software (Adobe Photoshop CS5).

### Hypoxia treatment *in vitro*

Primary rat mesenchymal cells, referred to as fibroblasts, were isolated from the ventral prostate of tumour-free Copenhagen rats as previously described^57^. Briefly, the prostate was removed, cut into small 1-mm cubes, washed with HBSS, and cultured in DMEM (Life Technologies) supplemented with 5% bovine serum (Life Technologies), 5% Nu-serum (BD Biosciences), 10 nM dihydrotestosterone, 5 μg/ml insulin, 100 units/ml penicillin and 100 μg/ml streptomycin (all Sigma). The explants were incubated at 37 °C, in an atmosphere of 5% CO_2_, and the medium was changed every other day. When the stromal cells that had migrated from the tissue were confluent, the explants were removed and the stromal cells passaged a few times before the start of the hypoxic incubation. Verification of a mesenchymal phenotype was done by immunohistochemical staining; showing that the cells were vimentin-positive (Atlas, HPA001762) and cytokeratin-5 and -18 negative (CK5, Covance, AF138 and CK18 Progen, GP-CK18). AT-1 and primary rat fibroblasts were grown in 6-well plates at 37 °C for 24 h under hypoxia (1.0% O_2,_ 5% CO_2_ and 94% N_2_) or under normoxia (21.0% O_2,_ 5% CO_2_ and 74% N_2_). Six replicates were used when total RNA was prepared, and *Lox* and *Loxl1-Loxl4* mRNA levels were examined as described above.

### MTT viability assay

Cell viability was determined with an MTT 3-(4,5-dimethylthiazol-2-yl)-2,5-diphenyltetrazolium bromide assay (product no. 11465007001, Roche Diagnostics). Briefly, AT-1 cells (10^3^ cells/well) were seeded on a 96-well plate in complete medium and incubated in 37 °C over night. The following day, the indicated concentrations of BAPN (0, 1, 100 or 500 μM) or recombinant LOX protein (Origene) (0, 15, 150 or 300 ng/ml) were added to the cells (6 replicates/concentration/time-point) and the cells were incubated for 24–96 hours in normoxia or for 24–48 hours in hypoxia as described above. MTT labelling reagent and solubilisation solution was added at the different time-points and absorbance was measured on the following day at 550 nm with a reference wavelength of 650 nm according to the manufacturer’s protocol.

### Statistics

Values are presented as mean ± SEM and the Student t-test was used for comparison between groups. The level of statistical significance was defined as *p* < 0.05. Statistical analysis was performed using the SPSS software version 22.0.0 (SPSS Inc.).

## Additional Information

**How to cite this article**: Nilsson, M. *et al*. Inhibition of Lysyl Oxidase and Lysyl Oxidase-Like Enzymes Has Tumour-Promoting and Tumour-Suppressing Roles in Experimental Prostate Cancer. *Sci. Rep*. **6**, 19608; doi: 10.1038/srep19608 (2016).

## References

[b1] Jansen M. K. & Csiszar K. Intracellular localization of the matrix enzyme lysyl oxidase in polarized epithelial cells. Matrix Biol 26, 136–139 (2007). 17074474 10.1016/j.matbio.2006.09.004 PMC1851931

[b2] Kagan H. M. & Li W. Lysyl oxidase: properties, specificity, and biological roles inside and outside of the cell. J Cell Biochem 88, 660–672 (2003). 12577300 10.1002/jcb.10413

[b3] Nellaiappan K., Risitano A., Liu G., Nicklas G. & Kagan H. M. Fully processed lysyl oxidase catalyst translocates from the extracellular space into nuclei of aortic smooth-muscle cells. J Cell Biochem 79, 576–582 (2000). 10996848 10.1002/1097-4644(20001215)79:4<576::aid-jcb60>3.0.co;2-a

[b4] Barker H. E., Cox T. R. & Erler J. T. The rationale for targeting the LOX family in cancer. Nat Rev Cancer 12, 540–552 (2012). 22810810 10.1038/nrc3319

[b5] Levental K. R. . Matrix crosslinking forces tumor progression by enhancing integrin signaling. Cell 139, 891–906 (2009). 19931152 10.1016/j.cell.2009.10.027 PMC2788004

[b6] Erler J. T. . Lysyl oxidase is essential for hypoxia-induced metastasis. Nature 440, 1222–1226 (2006). 16642001 10.1038/nature04695

[b7] Kirschmann D. A. . A molecular role for lysyl oxidase in breast cancer invasion. Cancer Res 62, 4478–4483 (2002). 12154058

[b8] Miller B. W. . Targeting the LOX/hypoxia axis reverses many of the features that make pancreatic cancer deadly: inhibition of LOX abrogates metastasis and enhances drug efficacy. EMBO Mol Med 7, 1063–1076 (2015). 26077591 10.15252/emmm.201404827 PMC4551344

[b9] Erler J. T. . Hypoxia-induced lysyl oxidase is a critical mediator of bone marrow cell recruitment to form the premetastatic niche. Cancer Cell 15, 35–44 (2009). 19111879 10.1016/j.ccr.2008.11.012 PMC3050620

[b10] Cox T. R. . The hypoxic cancer secretome induces pre-metastatic bone lesions through lysyl oxidase. Nature 522, 106–110 (2015). 26017313 10.1038/nature14492 PMC4961239

[b11] Barker H. E., Bird D., Lang G. & Erler J. T. Tumor-secreted LOXL2 activates fibroblasts through FAK signaling. Mol Cancer Res 11, 1425–1436 (2013). 24008674 10.1158/1541-7786.MCR-13-0033-T PMC3833835

[b12] Barry-Hamilton V. . Allosteric inhibition of lysyl oxidase-like-2 impedes the development of a pathologic microenvironment. Nat Med 16, 1009–1017 (2010). 20818376 10.1038/nm.2208

[b13] Barker H. E. . LOXL2-mediated matrix remodeling in metastasis and mammary gland involution. Cancer Res 71, 1561–1572 (2011). 21233336 10.1158/0008-5472.CAN-10-2868 PMC3842018

[b14] Wong C. C. . Lysyl oxidase-like 2 is critical to tumor microenvironment and metastatic niche formation in hepatocellular carcinoma. Hepatology 60, 1645–1658 (2014). 25048396 10.1002/hep.27320

[b15] Li R. K. . Lysyl oxidase-like 4 (LOXL4) promotes proliferation and metastasis of gastric cancer via FAK/Src pathway. J Cancer Res Clin Oncol 141, 269–281 (2015). 25216702 10.1007/s00432-014-1823-z PMC11823919

[b16] Deng H. . miR-193a-3p regulates the multi-drug resistance of bladder cancer by targeting the LOXL4 gene and the Oxidative Stress pathway. Mol Cancer 13, 234 (2014). 25311867 10.1186/1476-4598-13-234 PMC4200202

[b17] Contente S., Kenyon K., Rimoldi D. & Friedman R. M. Expression of gene rrg is associated with reversion of NIH 3T3 transformed by LTR-c-H-ras. Science 249, 796–798 (1990). 1697103 10.1126/science.1697103

[b18] Kenyon K. . Lysyl oxidase and rrg messenger RNA. Science 253, 802 (1991). 1678898 10.1126/science.1678898

[b19] Trackman P. C., Bedell-Hogan D., Tang J. & Kagan H. M. Post-translational glycosylation and proteolytic processing of a lysyl oxidase precursor. J Biol Chem 267, 8666–8671 (1992). 1349020

[b20] Uzel M. I. . Multiple bone morphogenetic protein 1-related mammalian metalloproteinases process pro-lysyl oxidase at the correct physiological site and control lysyl oxidase activation in mouse embryo fibroblast cultures. J Biol Chem 276, 22537–22543 (2001). 11313359 10.1074/jbc.M102352200

[b21] Palamakumbura A. H. . The propeptide domain of lysyl oxidase induces phenotypic reversion of ras-transformed cells. J Biol Chem 279, 40593–40600 (2004). 15277520 10.1074/jbc.M406639200

[b22] Palamakumbura A. H. . Lysyl oxidase propeptide inhibits prostate cancer cell growth by mechanisms that target FGF-2-cell binding and signaling. Oncogene 28, 3390–3400 (2009). 19597471 10.1038/onc.2009.203 PMC2753565

[b23] Agra N., Cidre F., Garcia-Garcia L., de la Parra J. & Alonso J. Lysyl oxidase is downregulated by the EWS/FLI1 oncoprotein and its propeptide domain displays tumor supressor activities in Ewing sarcoma cells. PLoS One 8, e66281 (2013). 23750284 10.1371/journal.pone.0066281 PMC3672102

[b24] Woznick A. R. . Lysyl oxidase expression in bronchogenic carcinoma. Am J Surg 189, 297–301 (2005). 15792754 10.1016/j.amjsurg.2004.11.031

[b25] Sung F. L. . Silencing of hypoxia-inducible tumor suppressor lysyl oxidase gene by promoter methylation activates carbonic anhydrase IX in nasopharyngeal carcinoma. Am J Cancer Res 4, 789–800 (2014). 25520868 PMC4266712

[b26] Payne S. L., Hendrix M. J. & Kirschmann D. A. Paradoxical roles for lysyl oxidases in cancer–a prospect. J Cell Biochem 101, 1338–1354 (2007). 17471532 10.1002/jcb.21371

[b27] Wu G. . LOXL1 and LOXL4 are epigenetically silenced and can inhibit ras/extracellular signal-regulated kinase signaling pathway in human bladder cancer. Cancer Res 67, 4123–4129 (2007). 17456585 10.1158/0008-5472.CAN-07-0012

[b28] Lapointe J. . Gene expression profiling identifies clinically relevant subtypes of prostate cancer. Proc Natl Acad Sci USA 101, 811–816 (2004). 14711987 10.1073/pnas.0304146101 PMC321763

[b29] Stewart G. D. . Analysis of hypoxia-associated gene expression in prostate cancer: lysyl oxidase and glucose transporter-1 expression correlate with Gleason score. Oncol Rep 20, 1561–1567 (2008). 19020742

[b30] Ren C., Yang G., Timme T. L., Wheeler T. M. & Thompson T. C. Reduced lysyl oxidase messenger RNA levels in experimental and human prostate cancer. Cancer Res 58, 1285–1290 (1998). 9515817

[b31] Pascal L. E. . Gene expression relationship between prostate cancer cells of Gleason 3, 4 and normal epithelial cells as revealed by cell type-specific transcriptomes. BMC Cancer 9, 452 (2009). 20021671 10.1186/1471-2407-9-452 PMC2809079

[b32] Ross A. E. . Gene expression pathways of high grade localized prostate cancer. Prostate 71, 1568–1577 (2011). 21360566 10.1002/pros.21373

[b33] Schmidt H. . [Mapping of a deletion interval on 8p21-22 in prostate cancer by gene dosage PCR]. Verh Dtsch Ges Pathol 91, 302–307 (2007). 18314628

[b34] Nilsson M. . High Lysyl Oxidase (LOX) in the Non-Malignant Prostate Epithelium Predicts a Poor Outcome in Prostate Cancer Patient Managed by Watchful Waiting. PLoS One 10, e0140985 (2015). 26501565 10.1371/journal.pone.0140985 PMC4621025

[b35] Adamo H. H., Halin Bergstrom S. & Bergh A. Characterization of a Gene Expression Signature in Normal Rat Prostate Tissue Induced by the Presence of a Tumor Elsewhere in the Organ. PLOS One 10, e0130076 (2015). 26076453 10.1371/journal.pone.0130076 PMC4468243

[b36] Tang S. S., Trackman P. C. & Kagan H. M. Reaction of aortic lysyl oxidase with beta-aminopropionitrile. J Biol Chem 258, 4331–4338 (1983). 6131892

[b37] Trackman P. C. & Kagan H. M. Nonpeptidyl amine inhibitors are substrates of lysyl oxidase. J Biol Chem 254, 7831–7836 (1979). 38246

[b38] Jung S. T., Kim M. S., Seo J. Y., Kim H. C. & Kim Y. Purification of enzymatically active human lysyl oxidase and lysyl oxidase-like protein from Escherichia coli inclusion bodies. Protein Expr Purif 31, 240–246 (2003). 14550642 10.1016/s1046-5928(03)00217-1

[b39] Kim M. S. . Expression and purification of enzymatically active forms of the human lysyl oxidase-like protein 4. J Biol Chem 278, 52071–52074 (2003). 14551188 10.1074/jbc.M308856200

[b40] Lee J. E. & Kim Y. A tissue-specific variant of the human lysyl oxidase-like protein 3 (LOXL3) functions as an amine oxidase with substrate specificity. J Biol Chem 281, 37282–37290 (2006). 17018530 10.1074/jbc.M600977200

[b41] Rodriguez H. M. . Modulation of lysyl oxidase-like 2 enzymatic activity by an allosteric antibody inhibitor. J Biol Chem 285, 20964–20974 (2010). 20439985 10.1074/jbc.M109.094136 PMC2898315

[b42] Halin S., Hammarsten P., Wikstrom P. & Bergh A. Androgen-insensitive prostate cancer cells transiently respond to castration treatment when growing in an androgen-dependent prostate environment. Prostate 67, 370–377 (2007). 17192959 10.1002/pros.20473

[b43] Bignon M. . Lysyl oxidase-like protein-2 regulates sprouting angiogenesis and type IV collagen assembly in the endothelial basement membrane. Blood 118, 3979–3989 (2011). 21835952 10.1182/blood-2010-10-313296

[b44] Denko N. C. . Investigating hypoxic tumor physiology through gene expression patterns. Oncogene 22, 5907–5914 (2003). 12947397 10.1038/sj.onc.1206703

[b45] Wong C. C. . Hypoxia-inducible factor 1 is a master regulator of breast cancer metastatic niche formation. Proc Natl Acad Sci USA 108, 16369–16374 (2011). 21911388 10.1073/pnas.1113483108 PMC3182724

[b46] Zenkel M. . Regulation of lysyl oxidase-like 1 (LOXL1) and elastin-related genes by pathogenic factors associated with pseudoexfoliation syndrome. Invest Ophthalmol Vis Sci 52, 8488–8495 (2011). 21948647 10.1167/iovs.11-8361

[b47] Kakkad S. M. . Hypoxic tumor microenvironments reduce collagen I fiber density. Neoplasia 12, 608–617 (2010). 20689755 10.1593/neo.10344 PMC2915405

[b48] Bondareva A. . The lysyl oxidase inhibitor, beta-aminopropionitrile, diminishes the metastatic colonization potential of circulating breast cancer cells. PLoS One 4, e5620 (2009). 19440335 10.1371/journal.pone.0005620 PMC2680032

[b49] Burns-Cox N., Avery N. C., Gingell J. C. & Bailey A. J. Changes in collagen metabolism in prostate cancer: a host response that may alter progression. J Urol 166, 1698–1701 (2001). 11586205 10.1016/s0022-5347(05)65656-x

[b50] Ayala G. . Reactive stroma as a predictor of biochemical-free recurrence in prostate cancer. Clin Cancer Res 9, 4792–4801 (2003). 14581350

[b51] Ahn S. G. . LOXL2 expression is associated with invasiveness and negatively influences survival in breast cancer patients. Breast Cancer Res Treat 141, 89–99 (2013). 23933800 10.1007/s10549-013-2662-3 PMC6944271

[b52] Gorogh T. . Selective upregulation and amplification of the lysyl oxidase like-4 (LOXL4) gene in head and neck squamous cell carcinoma. J Pathol 212, 74–82 (2007). 17354256 10.1002/path.2137

[b53] Peinado H. . A molecular role for lysyl oxidase-like 2 enzyme in snail regulation and tumor progression. EMBO J 24, 3446–3458 (2005). 16096638 10.1038/sj.emboj.7600781 PMC1276164

[b54] Moon H. J. . MCF-7 cells expressing nuclear associated lysyl oxidase-like 2 (LOXL2) exhibit an epithelial-to-mesenchymal transition (EMT) phenotype and are highly invasive *in vitro*. J Biol Chem 288, 30000–30008 (2013). 24014025 10.1074/jbc.C113.502310 PMC3798469

[b55] Isaacs J. T., Isaacs W. B., Feitz W. F. & Scheres J. Establishment and characterization of seven Dunning rat prostatic cancer cell lines and their use in developing methods for predicting metastatic abilities of prostatic cancers. Prostate 9, 261–281 (1986). 3774632 10.1002/pros.2990090306

[b56] Junqueira L. C., Bignolas G. & Brentani R. R. Picrosirius staining plus polarization microscopy, a specific method for collagen detection in tissue sections. Histochem J 11, 447–455 (1979). 91593 10.1007/BF01002772

[b57] Tuxhorn J. A. . Reactive stroma in human prostate cancer: induction of myofibroblast phenotype and extracellular matrix remodeling. Clin Cancer Res 8, 2912–2923 (2002). 12231536

